# Estimation of Canine *Leishmania* Infection Prevalence in Six Cities of the Algerian Littoral Zone Using a Bayesian Approach

**DOI:** 10.1371/journal.pone.0117313

**Published:** 2015-03-20

**Authors:** Amel Adel, Emmanuel Abatih, Niko Speybroeck, Abdelkrim Soukehal, Rachid Bouguedour, Karim Boughalem, Abdelmalek Bouhbal, Mouloud Djerbal, Claude Saegerman, Dirk Berkvens

**Affiliations:** 1 Institute of Veterinary Sciences, University Saad Dahlab, Blida, Algeria; 2 Institute of Tropical Medicine, Department of Biomedical Sciences, Nationalestraat 155, Antwerpen, Belgium; 3 Research Unit of Epidemiology and Risk Analysis applied to Veterinary Science (UREAR-ULg), Fundamental and Applied Research for Animals & Health (FARAH), Faculty of Veterinary Medicine, University of Liège, Boulevard de Colonster 20 B42, Sart-Tilman Liège, Belgium; 4 Université Catholique de Louvain, IRSS-FSP, Clos Chapelle aux Champs 30, Bruxelles, Belgium; 5 University Hospital of Beni Messous, Algiers, Algeria; 6 OIE Sub-Regional Representation for North Africa, 17 Avenue d’Afrique, El Menzah V 2091, Tunis, Tunesia; 7 Direction des Services Vétérinaires, Ministère de l’Agriculture et du Développement Rural, 12 bd Colonel Amirouche, 16000 Algiers, Algeria; 8 Regional Veterinary Laboratory of Draa-Ben-Kheda, Tizi-Ouzou, Algeria; Royal Tropical Institute, NETHERLANDS

## Abstract

A large-scale study on canine *Leishmania* infection (CanL) was conducted in six localities along a west-east transect in the Algerian littoral zone (Tlemcen, Mostaganem, Tipaza, Boumerdes, Bejaia, Jijel) and covering two sampling periods. In total 2,184 dogs were tested with an indirect fluorescent antibody test (IFAT) and a direct agglutination test (DAT). Combined multiple-testing and several statistical methods were compared to estimate the CanL true prevalence and tests characteristics (sensitivity and specificity). The Bayesian full model showed the best fit and yielded prevalence estimates between 11% (Mostaganem, first period) and 38% (Bejaia, second period). Sensitivity of IFAT varied (in function of locality) between 86% and 88% while its specificity varied between 65% and 87%. DAT was less sensitive than IFAT but showed a higher specificity (between 80% and 95% in function of locality or/and season). A general increasing trend of the CanL prevalence was noted from west to east. A concordance between the present results and the incidence of human cases of visceral leishmaniasis was observed, where also a maximum was recorded for Bejaia. The results of the present study highlight the dangers when using IFAT as a gold standard.

## Introduction

Leishmaniasis caused by *Leishmania infantum* is endemic in the Mediterranean basin where the dog is considered the main domestic reservoir for human visceral leishmaniasis (VL) [1].

Canine leishmaniasis (CanL) caused by *L. infantum* is a severe zoonotic disease that affects millions of dogs [2]. The parasites are transmitted by the bites of female sandflies of the genus *Phlebotomus* (Phlebotominae, Diptera) that are in Algeria *Phlebotomus perniciosus* and *P. longicuspis* [3, 4]. The incubation period of CanL ranges from a few months to several years [5]. The clinical features of the disease vary from subclinical self-limiting infection to fatal disease [5, 6].

Both diseased and sub-clinically infected dogs are infectious to sand fly vectors, allowing transmission of the parasite to other dogs or humans [7, 8]. That is why prompt diagnosis of infected dogs is essential. Microscopic examination of smears of lymph node and bone marrow aspirates, along with serology and polymerase chain reaction, are the most frequently used diagnostic methods for CanL [9, 10]. However, according to the World Organisation for Animal Health serology is the preferred method for diagnosis of CanL and VL, even during the early stages of the disease: with a purported sensitivity of 96% and a purported specificity of 98%, immunofluorescence antibody test (IFAT) is considered the most suited test for field diagnosis [11].

A first epidemiological survey involving 462 dogs of Algiers, in which three serological tests were assessed in different dog categories, showed that IFAT was the most sensitive. However, its specificity was considerably lower in farm dogs (65%) [12]. Therefore, in the present study, IFAT was combined with the direct agglutination test (DAT) to carry out a cross-sectional survey in six cities of the sea coast region of Algeria. This area was chosen because the geographical distribution of VL covers all the humid and sub-humid regions in the north of the country [13–16].

Several statistical methods have been developed to assess the true prevalence and the diagnostic accuracy in the absence of a true gold standard [17]. The true prevalence is the number of truly infected individuals of a tested population [18]. When no perfect test is available, the results will provide an estimate of the true prevalence known as the apparent prevalence. This is also called the seroprevalence when using serological diagnostic tests. Latent class models for which the true disease status is considered to be a latent variable were often used for estimating test accuracy and disease prevalence in the absence of a gold standard [19]. The first model suggested by Hui and Walter [20] is useful when two or more tests are applied to the same individuals from two or more populations. This method uses the maximum likelihood procedure under the assumption of conditional independence between test results, that sensitivity and specificity are unchanged in the two populations and that each population has distinct disease prevalence. If these assumptions are not met, we use a Bayesian approach to draw inferences about the disease prevalence and test properties while adjusting for the possibility of conditional dependence between tests [21]. Bayesian statistics is a theory for interweaving new and existing data by taking both sources into account [19].

The objective of the present study was to estimate the true prevalence of canL in a large dog population before and after the vector season, comparing the results obtained from standard frequentist approaches and different estimation models used in a Bayesian framework. At the same time, the diagnostic test characteristics of IFAT and DAT were also evaluated.

## Materials and Methods

### Ethics statement

Authorisation to conduct the survey was obtained from the Direction des Services Vétérinaires (DSV, Ministry of Agriculture) and the Institut National de Médecine Vétérinaire (INMV, Ministry of Agriculture). The protocol, including blood collection procedures, was approved by the Doctoral Committee at the Institute of Tropical Medicine, Antwerp and the Doctoral Committee of the Faculty of Veterinary Medicine at the University of Liège. Privately owned dogs were included in the study. Blood collection was done by means of cephalic veinal puncture, after minimal restraining and without anaesthetic. At each Wilaya (locality, township), the study was authorised and supervised by the respective Inspection Vétérinaire de Wilaya (IVW de Tlemcen, Mostaganem, Tipaza, Boumerdes, Jijel and Bejaia), operating under the umbrella of the Direction des Services Vétérinaires. The official veterinary officer of the Inspection Vétérinaire de Wilaya in question carried out the blood collection after obtaining oral permission from the dog owner. The study took place on the territories of the six aforementioned Wilayate (Tlemcen, Mostaganem, Tipaza, Boumerdes, Jijel and Bejaia). The field studies did not involve endangered or protected species.

### Study area

**Fig 1:**
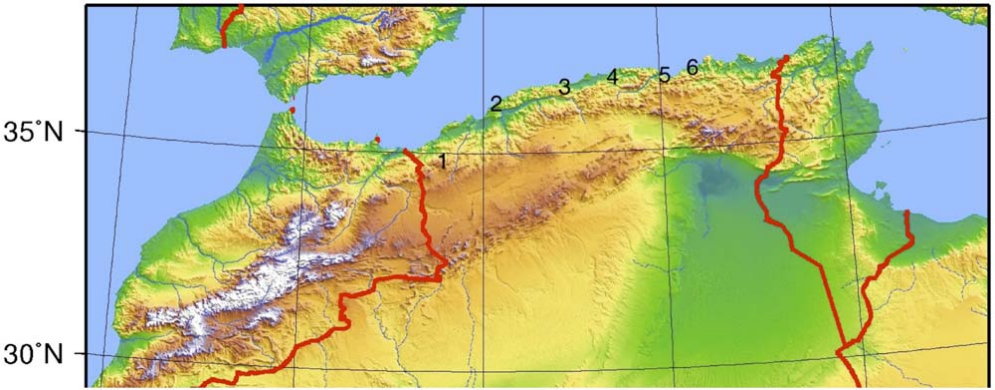
Location of localities sampled. 1 = Tlemcen, 2 = Mostaganem, 3 = Tipaza, 4 = Boumerdes, 5 = Bejaia, 6 = Jijel.

A cross-sectional study was conducted in six Wilayate (singular: Wilaya = locality) of the Algerian littoral zone (from west to east): Tlemcen, Mostaganem, Tipaza, Boumerdes, Bejaia and Jijel (Fig. 1). The original list also included Oran and Annaba, but no collaboration could be established there because of a rabies scare. Within the selected localities, surveys were carried out between February and April 2008 (first phase, before the vector season, which extends from May to October) and between November 2008 and February 2009 (second phase, after the vector season) [15].

### Animals

A random sample of the canine population of all municipalities at each location was obtained in each collection phase. No distinction between sex and age was made. No stray dogs were included as there were no kennels in the localities included in the sampling frame. With a maximum prevalence of 11% estimated in Algiers within the group of stray dogs [12] and an absolute precision of 5%, the sample size was 150 dogs. Thus, we requested the veterinary officers to collect 200 samples in each city in each season. All animals were in judged to be in good health.

### Serology

Blood from the cephalic vein was collected in dry labelled tubes. Two serological tests were used: an indirect immunofluorescence antibody test (IFAT) and a direct agglutination test (DAT). Both tests were selected after evaluation in different groups of dogs in Algiers [12]: IFAT showed a high sensitivity in all groups(⩾ 90%), but a low specificity in farm dogs (65%), whereas DAT had a high specificity in all groups (⩾ 85%).

IFAT was performed according to [22] and [23]. The antigen consisted of promastigotes of *L. infantum*. Anti-*Leishmania* antibodies were detected by secondary antibodies against rabbits, anti-dog immunoglobulins G (IgG) conjugated with fluorescein isothiacyanate (FITC) (Sigma-Aldrich, St Louis, MO, USA). A 1:128 dilution was used as cut-off value [24, 25].

DAT was performed using a commercial kit, available from the Institute of Tropical Medicine (Antwerp). A dilution of 1:320 was used as cut-off value [26, 27].

### Statistical analysis

The data generated by the survey produce 12 sets (six localities times two sampling periods) of four data points each (Table 1).

**Table 1:** Data structure per locality per sampling round. $T_{i}^{j}$: number of test results with *i* = test (1 = IFAT, 2 = DAT) and *j* = test result (– = negative, + = positive).

		DAT	
		-ve	+ve
IFAT	-ve	$T_{1}^{-}T_{2}^{-}$	$T_{1}^{-}T_{2}^{+}$
	+ve	$T_{1}^{+}T_{2}^{-}$	$T_{1}^{+}T_{2}^{+}$

The apparent prevalence (laboratory seroprevalence, proportion positive test results) per test and combination of tests: parallel interpretation (at least one positive test result signifies a positive dog) and serial interpretation (both tests must be positive to be a case) and their exact 95% confidence intervals were calculated [l_0.025_ = qbeta(0.025, n_positive_, n_negative_+1); l_0.975_ = qbeta(0.975, n_positive_+1, n_negative_) with qbeta being the beta quantile function with parameters (probability, shape1, shape2) and n_…_ the number of positive or negative test results]. True and apparent prevalence by diagnostic test and by sampling period were plotted on a map produced with QGIS 2.0.1, using georeferenced data on the administrative regions of Algeria (obtained from http://www.diva-gis.org/gdata).

Concordance between test results was expressed in terms of indices of positive and negative agreement [28] and their 95% confidence intervals were calculated according to [29]. Computations were done in R 3.1.1 (http://www.r-project.org). The code is shown in S1 Listing 1.

Estimation of the true prevalence and test characteristics was attempted in several ways. As explained in detail in [30], a full two-diagnostic-test model, assuming no conditional independence between the two tests, consists of three independent equations with seven variables to estimate (refer to Table 1 for T_i_^+/-^; D^+^ D^-^ refer to respectively truly infected and truly infection-free dogs):

$$\begin{array}{cc} p(T_{1}^{+}T_{2}^{+}) & =\vartheta_{1}\vartheta_{2}\vartheta_{4}+(1-\vartheta_{1})(1-\vartheta_{3})(1-\vartheta_{7})\\ p(T_{1}^{+}T_{2}^{-}) & =\vartheta_{1}\vartheta_{2}(1-\vartheta_{4})+(1-\vartheta_{1})(1-\vartheta_{3})\vartheta_{7}\\ p(T_{1}^{-}T_{2}^{+}) & =\vartheta_{1}(1-\vartheta_{2})\vartheta_{5}+(1-\vartheta_{1})\vartheta_{3}(1-\vartheta_{6})\\ p(T_{1}^{-}T_{2}^{-}) & =\vartheta_{1}(1-\vartheta_{2})(1-\vartheta_{5})+(1-\vartheta_{1})\vartheta_{3}\vartheta_{6}\\ & =1-p(T_{1}^{+}T_{2}^{+})-p(T_{1}^{+}T_{2}^{-})-p(T_{1}^{-}T_{2}^{+})\\ \vartheta_{1} & =p(D^{+})\;\;[p(D^{-})=1-\vartheta_{1}]\\ \vartheta_{2} & =p(T_{1}^{+}|D^{+})\\ \vartheta_{3} & =p(T_{1}^{-}|D^{-})\\ \vartheta_{4} & =p(T_{2}^{+}|D^{+}\cap T_{1}^{+})\\ \vartheta_{5} & =p(T_{2}^{+}|D^{+}\cap T_{1}^{-})\\ \vartheta_{6} & =p(T_{2}^{-}|D^{-}\cap T_{1}^{-})\\ \vartheta_{7} & =p(T_{2}^{-}|D^{-}\cap T_{1}^{+}) \end{array}$$

The current data set has 36 independent equations (six localities ×two periods ×three equations) and, assuming conditional test dependence and specific diagnostic test characteristics per locality per period, requires 84 variables to be estimated (six localities ×two periods ×seven variables). Because the number of variables to estimate exceeds the number of independent equations, prior deterministic or probabilistic constraints need to be applied to allow estimation [30].

The starting point is the simplified so-called Hui-Walter model Appendix [20], whereby tests are assumed conditionally independent and test sensitivity and test specificity are assumed constant over the two sampling periods per locality. Per locality, this model results in six independent equations (three for the first sampling period and three for the second) and six variables to be estimated (prevalence first period, prevalence second period and two sensitivities and specificities – note that this results in six variables ×six localities = 36 variables in total). This model is thus entirely driven by deterministic constraints. The starting model is (*p*_*i*_, *i* ∈ {1, 2} for respectively period 1 and period 2):

$$\begin{array}{cc} p_{1}(T_{1}^{+}T_{2}^{+}) & =\vartheta_{1}\vartheta_{3}\vartheta_{5}+(1-\vartheta_{1})(1-\vartheta_{4})(1-\vartheta_{8})\\ p_{1}(T_{1}^{+}T_{2}^{-}) & =\vartheta_{1}\vartheta_{3}(1-\vartheta_{5})+(1-\vartheta_{1})(1-\vartheta_{4})\vartheta_{8}\\ p_{1}(T_{1}^{-}T_{2}^{+}) & =\vartheta_{1}(1-\vartheta_{3})\vartheta_{7}+(1-\vartheta_{1})\vartheta_{4}(1-\vartheta_{6})\\ p_{1}(T_{1}^{-}T_{2}^{-}) & =\vartheta_{1}(1-\vartheta_{3})(1-\vartheta_{7})+(1-\vartheta_{1})\vartheta_{4}\vartheta_{6}\\ & =1-p_{1}(T_{1}^{+}T_{2}^{+})-p_{1}(T_{1}^{+}T_{2}^{-})-p_{1}(T_{1}^{-}T_{2}^{+})\\ p_{2}(T_{1}^{+}T_{2}^{+}) & =\vartheta_{2}\vartheta_{9}\vartheta_{11}+(1-\vartheta_{2})(1-\vartheta_{10})(1-\vartheta_{14})\\ p_{2}(T_{1}^{+}T_{2}^{-}) & =\vartheta_{2}\vartheta_{9}(1-\vartheta_{11})+(1-\vartheta_{2})(1-\vartheta_{10})\vartheta_{14}\\ p_{2}(T_{1}^{-}T_{2}^{+}) & =\vartheta_{2}(1-\vartheta_{9})\vartheta_{13}+(1-\vartheta_{2})\vartheta_{10}(1-\vartheta_{12})\\ p_{2}(T_{1}^{-}T_{2}^{-}) & =\vartheta_{2}(1-\vartheta_{9})(1-\vartheta_{13})+(1-\vartheta_{2})\vartheta_{10}\vartheta_{12}\\ & =1-p_{2}(T_{1}^{+}T_{2}^{+})-p_{2}(T_{1}^{+}T_{2}^{-})-p_{2}(T_{1}^{-}T_{2}^{+})\\ \vartheta_{1} & =p_{1}(D^{+})\;\;[p_{1}(D^{-})=1-\vartheta_{1}]\\ \vartheta_{2} & =p_{2}(D^{+})\;\;[p_{2}(D^{-})=1-\vartheta_{2}]\\ \vartheta_{3} & =p_{1}(T_{1}^{+}|D^{+});\vartheta_{9}=p_{2}(T_{1}^{+}|D^{+})\\ \vartheta_{4} & =p_{1}(T_{1}^{-}|D^{-});\vartheta_{10}=p_{2}(T_{1}^{-}|D^{-})\\ \vartheta_{5} & =p_{1}(T_{2}^{+}|D^{+}\cap T_{1}^{+});\vartheta_{11}=p_{2}(T_{2}^{+}|D^{+}\cap T_{1}^{+})\\ \vartheta_{6} & =p_{1}(T_{2}^{-}|D^{-}\cap T_{1}^{-});\vartheta_{12}=p_{2}(T_{2}^{-}|D^{-}\cap T_{1}^{-})\\ \vartheta_{7} & =p_{1}(T_{2}^{-}|D^{+}\cap T_{1}^{-});\vartheta_{13}=p_{2}(T_{2}^{+}|D^{+}\cap T_{1}^{-})\\ \vartheta_{8} & =p_{1}(T_{2}^{-}|D^{-}\cap T_{1}^{+});\vartheta_{14}=p_{1}(T_{2}^{-}|D^{-}\cap T_{1}^{+}) \end{array}$$

The Hui-Walter deterministic constraints are *ϑ*_3_ = *ϑ*_9_; *ϑ*_4_ = *ϑ*_10_; *ϑ*_5_ = *ϑ*_7_ = *ϑ*_11_ = *ϑ*_13_; *ϑ*_6_ = *ϑ*_8_ = *ϑ*_12_ = *ϑ*_14_ and the model thus becomes:

$$\begin{array}{cc} p_{1}(T_{1}^{+}T_{2}^{+}) & =\vartheta_{1}\vartheta_{3}\vartheta_{5}+(1-\vartheta_{1})(1-\vartheta_{4})(1-\vartheta_{6})\\ p_{1}(T_{1}^{+}T_{2}^{-}) & =\vartheta_{1}\vartheta_{3}(1-\vartheta_{5})+(1-\vartheta_{1})(1-\vartheta_{4})\vartheta_{6}\\ p_{1}(T_{1}^{-}T_{2}^{+}) & =\vartheta_{1}(1-\vartheta_{3})\vartheta_{5}+(1-\vartheta_{1})\vartheta_{4}(1-\vartheta_{6})\\ p_{1}(T_{1}^{-}T_{2}^{-}) & =\vartheta_{1}(1-\vartheta_{3})(1-\vartheta_{5})+(1-\vartheta_{1})\vartheta_{4}\vartheta_{6}\\ p_{2}(T_{1}^{+}T_{2}^{+}) & =\vartheta_{2}\vartheta_{3}\vartheta_{5}+(1-\vartheta_{2})(1-\vartheta_{4})(1-\vartheta_{6})\\ p_{2}(T_{1}^{+}T_{2}^{-}) & =\vartheta_{2}\vartheta_{3}(1-\vartheta_{5})+(1-\vartheta_{2})(1-\vartheta_{4})\vartheta_{6}\\ p_{2}(T_{1}^{-}T_{2}^{+}) & =\vartheta_{2}(1-\vartheta_{3})\vartheta_{5}+(1-\vartheta_{2})\vartheta_{4}(1-\vartheta_{6})\\ p_{2}(T_{1}^{-}T_{2}^{-}) & =\vartheta_{1}(1-\vartheta_{3})(1-\vartheta_{5})+(1-\vartheta_{1})\vartheta_{4}\vartheta_{6}\\ \vartheta_{1} & =p_{1}(D^{+})\;\;[p_{1}(D^{-})=1-\vartheta_{1}]\\ \vartheta_{2} & =p_{2}(D^{+})\;\;[p_{2}(D^{-})=1-\vartheta_{2}]\\ \vartheta_{3} & =p_{1}(T_{1}^{+}|D^{+})\\ \vartheta_{4} & =p_{1}(T_{1}^{-}|D^{-})\\ \vartheta_{5} & =p_{1}(T_{2}^{+}|D^{+})\\ \vartheta_{6} & =p_{1}(T_{2}^{-}|D^{-}) \end{array}$$

This model is run in WinBUGS [31]. The code is shown in S2 Listing 2. Model fit and concordance between prior constraints and data are assessed by means of *Bayes*−*p* value, *DIC* and *p*_*D*_ (see [30] for a detailed description of the use of these statistics). The prior constraints applied to the two prevalences are necessary because of the existence of two mirror-symmetric solutions when solving the Rogan-Gladen equation [32]:

$$p^{\prime }=pSe+(1-p)(1-Sp)$$

with:

$$\begin{array}{cc} p^{\prime } & =\text{apparent prevalence}\\ p & =\text{true prevalence}\\ \mathrm{Se} & =\text{test sensitivity}\\ \mathrm{Sp} & =\text{test specificity} \end{array}$$

Solutions are: {*p*_1_ = *p*; *Se*_1_ = *Se*; *Sp*_1_ = *Sp*} and {*p*_2_ = 1−*p*; *Se*_2_ = 1−*Sp*; *Sp*_2_ = 1−*Se*}. Applying a (realistic) prior uniform {0, 0.5} to *p* forces the solution towards {*p*_1_, *Se*_1_, *Sp*_1_}.

A second approach also limits the number of variables to be estimated to 36. Contrary to the Hui-Walter model, it allows for conditional test dependence and different test characteristics in different locality-period combinations. Reduction of 84 variables to be estimated to 36 is achieved by combining the localities into two groups, based on the level of apparent prevalence: a first group contains the two westernmost localities (Tlemcen and Mostaganem), the second group the four other localities (Tipaza, Boumerdes, Bejaia and Jijel). The 36 variables to be estimated are thus: 12 prevalences and 4 groups ×6 variables per group (*ϑ*_2_…*ϑ*_7_). The WinBUGS listing is shown in S3 Listing 3.

As stated before, the full model for the current data set, assuming conditional dependence and different variable values for the six localities ×two sampling periods combinations thus has 36 independent equations and 84 variables. It is obvious that this model is over-specified and that external information is required in order to obtain estimates for the variables. The external (prior) information was extracted from [12]: this article demonstrated that the diagnostic test characteristics (both for IFAT and DAT) depend on the type of dog on which the test is applied. The type of dog was not unequivocally established in the current survey, although most of the animals could be classified as either farm dog or guard dog. The limits used for the prior information were therefore the minimum value of the lower limits of the 95% confidence intervals obtained for farm dogs and guard dogs and the maximum value of the corresponding upper limits. *Bayes-p*, *DIC* and *p_D_* values were used to guide model adjustment, which in this case consisted mainly of refining the ranges of the prior uniform distributions. Since only values for sensitivity and specificity were available from [12], a model based on covariances was used to model conditional test dependence [21]. The WinBUGS listing of the basic model is shown in S4 Listing 4.

## Results

A total of 2,184 dogs were sampled during the two phases (1,180 during the first sampling period and 1,004 during the second). The detailed results per locality, period and test are given in Table 2.

**Table 2:** Contingency tables per locality per sampling period with positive (+ve) and negative (-ve) results in IFAT and DAT.

	Phase 1				Phase 2			
	IFAT	DAT			IFAT	DAT		
		-ve	+ve	Total		-ve	+ve	Total
Tlemcen	-ve	107	12	119	-ve	94	13	107
	+ve	47	16	63	+ve	29	16	45
	Total	154	28	182	Total	123	29	152
Mostaganem	-ve	81	0	81	-ve	120	3	123
	+ve	50	12	62	+ve	27	19	46
	Total	131	12	143	Total	147	22	169
Tipaza	-ve	111	49	160	-ve	123	19	142
	+ve	56	26	82	+ve	17	50	67
	Total	167	75	242	Total	140	69	209
Boumerdes	-ve	114	21	135	-ve	59	7	66
	+ve	54	17	71	+ve	17	24	41
	Total	168	38	206	Total	76	31	107
Bejaia	-ve	76	28	104	-ve	97	11	108
	+ve	64	30	94	+ve	32	63	95
	Total	140	58	198	Total	129	74	203
Jijel	-ve	117	30	147	-ve	96	7	103
	+ve	32	30	62	+ve	21	40	61
	Total	149	60	209	Total	117	47	164

**Fig 2:**
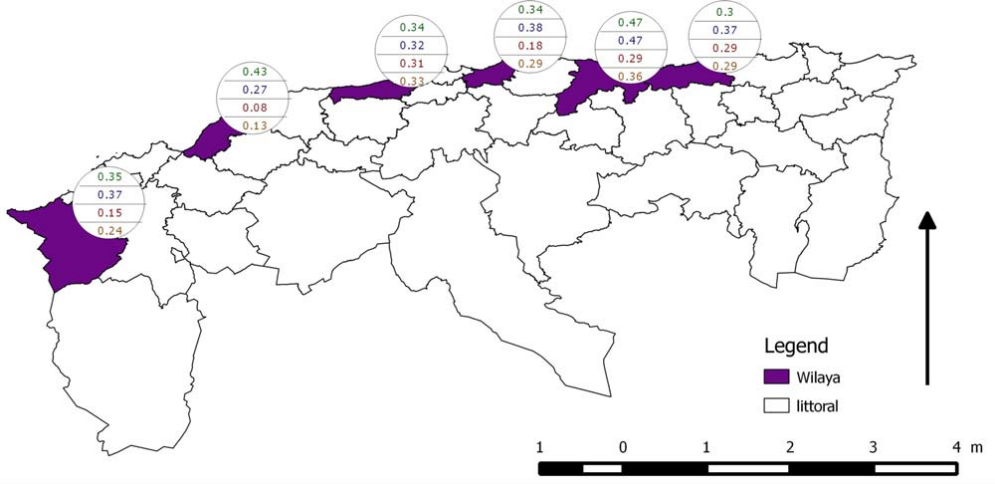
Apparent prevalence per test per sampling period per locality. From top to bottom: IFAT first period; IFAT second period; DAT first period; DAT second period.

**Fig 3:**
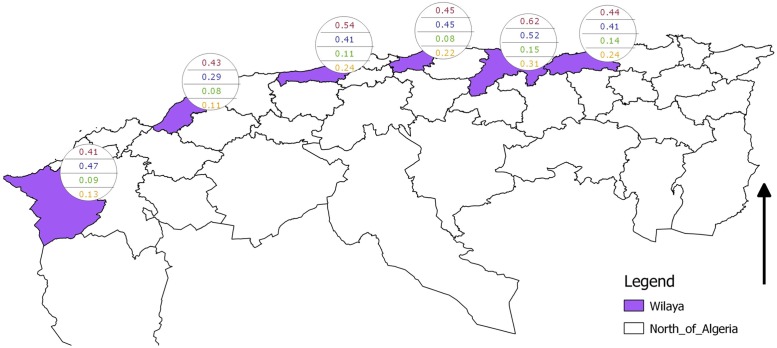
Apparent prevalence per locality. From top to bottom: only IFAT, only DAT, serial interpretation of the two test results and parallel interpretation of the two test results.

Table 3 and Figs. 2 and 3 show the apparent prevalences, the proportion positive results per locality and sampling round. IFAT-based apparent prevalence is nearly always higher than that based on DAT (the only exception is Tipaza during the second sampling round). Within each locality, IFAT-based apparent prevalence is very similar for the two sampling phases. The same is true for DAT-based seroprevalence, with the exception of Tlemcen, Boumerdes and Bejaia where higher values are observed during the second sampling round. Parallel and serial interpretation of the results are much more variable: there is no difference between the two sampling rounds for the serial interpretation based apparent prevalence for the two most western localities (Tlemcen and Mostaganem), but is higher during the second sampling round for the four more eastern localities.

**Table 3:** Apparent prevalences with 95% confidence interval per locality, sampling phase and test: IFAT = indirect immunofluorescence test; DAT = direct agglutination test; PAR = parallel interpretation of the two test results; SER = serial interpretation of the two test results.

		First round	Second round
Tlemcen	IFAT	0.35 (0.28–0.42)	0.37 (0.28–0.46)
	DAT	0.15 (0.10–0.21)	0.24 (0.16–0.32)
	PAR	0.41 (0.34–0.49)	0.47 (0.38–0.56)
	SER	0.09 (0.05–0.14)	0.13 (0.08–0.20)
Mostaganem	IFAT	0.43 (0.35–0.52)	0.27 (0.21–0.35)
	DAT	0.08 (0.04–0.14)	0.13 (0.08–0.19)
	PAR	0.43 (0.35–0.52)	0.29 (0.22–0.36)
	SER	0.08 (0.04–0.14)	0.11 (0.07–0.17)
Tipaza	IFAT	0.34 (0.28–0.40)	0.32 (0.26–0.39)
	DAT	0.31 (0.25–0.37)	0.33 (0.27–0.40)
	PAR	0.54 (0.47–0.61)	0.41 (0.34–0.48)
	SER	0.11 (0.07–0.15)	0.24 (0.18–0.30)
Boumerdes	IFAT	0.34 (0.28–0.41)	0.38 (0.29–0.48)
	DAT	0.18 (0.13–0.24)	0.29 (0.21–0.39)
	PAR	0.45 (0.38–0.52)	0.45 (0.35–0.55)
	SER	0.08 (0.05–0.13)	0.22 (0.15–0.32)
Bejaia	IFAT	0.47 (0.40–0.55)	0.47 (0.40–0.54)
	DAT	0.29 (0.23–0.36)	0.36 (0.30–0.43)
	PAR	0.62 (0.54–0.68)	0.52 (0.45–0.59)
	SER	0.15 (0.10–0.21)	0.31 (0.25–0.38)
Jijel	IFAT	0.30 (0.24–0.36)	0.37 (0.30–0.45)
	DAT	0.29 (0.23–0.35)	0.29 (0.22–0.36)
	PAR	0.44 (0.37–0.51)	0.41 (0.34–0.49)
	SER	0.14 (0.10–0.20)	0.24 (0.18–0.31)

Indices of positive and negative agreement and their confidence intervals are shown in Table 4: the overall index of positive agreement was 0.52 (95% CI: 0.48–0.55) and the overall index of negative agreement was 0.79 (95% CI: 0.77–0.80).

**Table 4:** Concordance between IFAT and DAT: indices of positive agreement (IPA) and negative agreement (INA) and 95% confidence intervals.

		First round	Second round
Tlemcen	IPA	0.35 (0.23–0.48)	0.43 (0.29–0.57)
	INA	0.78 (0.73–0.84)	0.81 (0.76–0.87)
Mostaganem	IPA	0.32 (0.20–0.45)	0.56 (0.42–0.69)
	INA	0.76 (0.69–0.82)	0.88 (0.84–0.92)
Tipaza	IPA	0.33 (0.24–0.43)	0.74 (0.63–0.80)
	INA	0.68 (0.61–0.73)	0.87 (0.83–0.90)
Boumerdes	IPA	0.31 (0.20–0.43)	0.67 (0.54–0.77)
	INA	0.75 (0.70–0.80)	0.83 (0.76–0.89)
Bejaia	IPA	0.39 (0.30–0.49)	0.75 (0.67–0.81)
	INA	0.62 (0.55–0.70)	0.81 (0.76–0.87)
Jijel	IPA	0.49 (0.39–0.59)	0.74 (0.64–0.82)
	INA	0.79 (0.73–0.83)	0.87 (0.82–0.91)
Overall	IPA	0.37 (0.33–0.42)	0.68 (0.64–0.72)
	INA	0.73 (0.71–0.75)	0.85 (0.83–0.87)

Three trends are observed in the indices of agreement: (i) indices of negative agreement are invariably higher than those of positive agreement, (ii) indices of agreement are higher during the second sampling period than during the first period, (iii) there is an increase in indices of positive agreement along the west-east transect.

**Fig 4:**
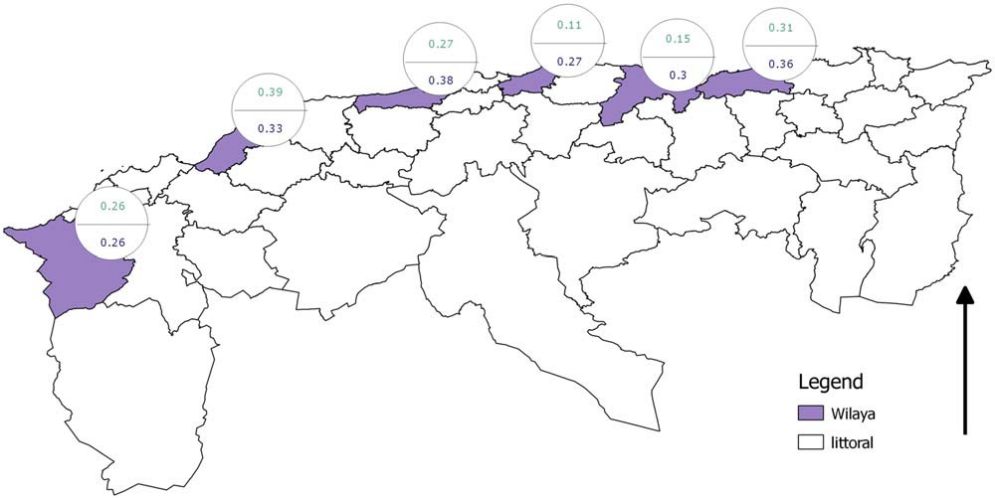
Canine *Leishmania* infection prevalence estimates according to the Hui-Walter model. Top prevalence is for first sampling period, bottom for second.

The results of the Hui-Walter model are shown in Table 5 and Fig. 4. With the exception of Tlemcen, *Bayes-p* values for all localities lie close to unity, indicating poor model fit. At Tlemcen, the *p_D_* value for the first period, calculated from the posterior means of the variable nodes is markedly smaller than that calculated from the mean posterior probabilities, again indicating lack of concordance between prior information and data [30]. The same lack of fit is observed for the model, based on the grouped localities: the overall *p_D_* equals -14.1 and the *Bayes-p* values vary between 0.41 and 0.91.

**Table 5:** Estimates from the Hui-Walter model.

Locality	Pr_1_	Pr_2_	Se_FAT_	Sp_IFAT_	Se_DAT_	Sp_DAT_	*Bayes-p*	*p_D_*	*DIC*
Tlemcen	0.26	0.26	0.72	0.81	0.52	0.92	0.51, 0.56	0.49, 2.62 (2.13, 2.08)	17.6, 19.7 (19.3, 19.3)
Mostaganem	0.39	0.33	0.63	0.72	0.24	0.92	0.95, 0.89	-9.9, -28.7 (1.74, 2.45)	-4.5, 6.4 (20.9, 22.2)
Tipaza	0.27	0.38	0.32	0.57	0.33	0.59	0.98, 0.99	4.06,-56.12 (3.31, 3.09)	34.2, -16.1 (33.52, 40.73)
Boumerdes	0.11	0.27	0.83	0.73	0.80	0.88	0.58, 0.85	1.66, 2.48 (2.52, 1.99)	20.3, 22.5 (21.1, 22.0)
Bejaia	0.15	0.30	0.93	0.66	0.90	0.83	0.95, 0.99	1.58, 2.52 (2.48, 2.01)	28.3, 33.2 (29.1, 32.7)
Jijel	0.31	0.36	0.59	0.70	0.53	0.74	0.75, 0.97	-14.26, -44.69 (2.85, 2.04)	6.68, -18.03 (23.8, 28.7)

Pr_1_ = prevalence first period; Pr_2_ = prevalence second period; Se_IFAT_ = sensitivity IFAT; Sp_IFAT_ = specificity IFAT; Se_DAT_ = sensitivity DAT; Sp_DAT_ = specificity DAT; *p_D_*, *DIC* top line = values calculated from posterior values of variable nodes resp. for first period and second period, (bottom line) = values calculated from posterior probabilities

**Fig 5:**
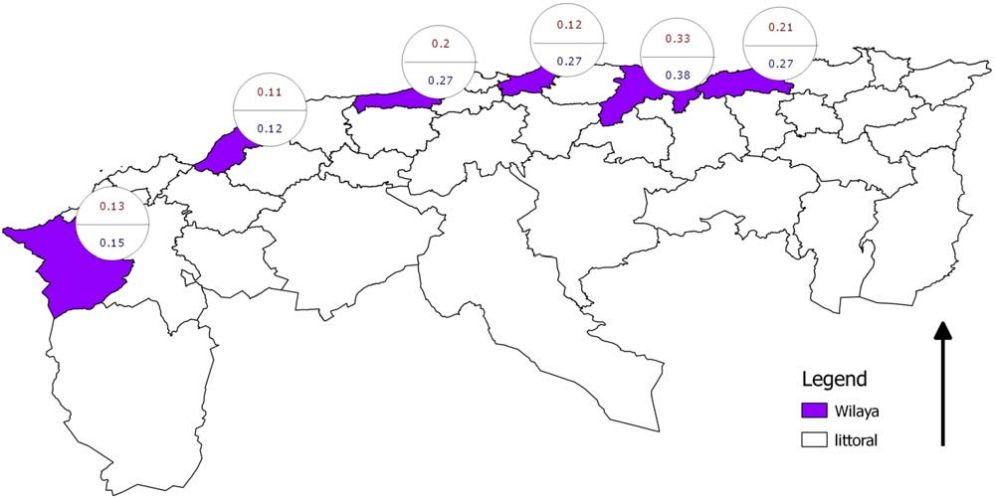
Canine *Leishmania* infection prevalence estimates according to full conditional test dependence model. Top prevalence is for first sampling period, bottom for second.

The full model did result in a good fit (*p_D_* = 27.92, *DIC* = 234.52 and Bayes-p values between 0.47 and 0.72) and yielded significant covariances between specificities, whose posterior probability distributions did not include zero, indicating conditional dependence. The estimates obtained from this model are shown in Table 6 and Fig. 5. The results indicate an increase in prevalence from west to east and an increase in prevalence from the first survey period to the second, although credibility intervals overlap. Also noteworthy is the increase in IFAT specificity from the first survey period to the second, although the same remark about credibility intervals applies.

**Table 6:** Canine *Leishmania* infection true prevalence and diagnostic test characteristics estimated from the full conditional dependence model.

				IFAT		DAT	
Period	Locality	Prevalence		Sensitivity	Specificity	Sensitivity	Specificity
1	Tlemcen	0.13	a^†^	0.87	0.73	0.56	0.89
1	Mostaganem	0.11	a	0.88	0.65	0.57	0.95
1	Tipaza	0.20	ab	0.86	0.77	0.64	0.80
1	Boumerdes	0.12	a	0.87	0.73	0.56	0.85
1	Bejaia	0.33	c	0.86	0.72	0.54	0.82
1	Jijel	0.21	ab	0.86	0.85	0.65	0.82
2	Tlemcen	0.15	a	0.87	0.80	0.57	0.86
2	Mostaganem	0.12	a	0.87	0.80	0.59	0.91
2	Tipaza	0.27	bc	0.86	0.87	0.78	0.84
2	Boumerdes	0.27	bc	0.87	0.80	0.67	0.85
2	Bejaia	0.38	c	0.87	0.79	0.73	0.86
2	Jijel	0.27	bc	0.87	0.81	0.70	0.86

^†^lines with the same letter are not different from one another

## Discussion

The apparent prevalence obtained with IFAT varied between 30% (Jijel) and 47% (Bejaia) during the first phase and from 27% (Mostaganem) to 47% (Bejaia) during the post-vectorial phase, while the estimate with DAT was lower (8% at Mostaganem to 31% at Tipaza for the first phase and 13% at Mostaganem to 36% at Bejaia during the second). These estimates lie towards the higher end of the range of results reported for other countries in the mediterranean basin using IFAT: Tunisia 18-53% [33, 34]; Morocco 9-19% [35, 36]; Malaga (Spain) 35% [37]; western Liguria (Italy) 30% [38]; Corfu (Greece) 50% [39]. Results, obtained when using DAT, ranged from 6% in Portugal [40] to 27% in Corsica [41] to 28% in Turkey with a 100% agreement with IFAT results [42].

IFAT is recommended as a reference test (best available practicable method to determine the animal’s true infection status) by the World Organization for Animal Health [11]. However, a study conducted in Algiers, Algerian capital and in the littoral zone [12], demonstrated the variability of test characteristics (sensitivity and specificity) in different dog populations: IFAT was highly sensitive in all types of dogs and very specific in stray dogs, but was shown to be less specific in farm dogs, whereas DAT maintained a relatively high specificity in all groups. Chaouch and colleagues [43] examined 75 dogs in Tunis, suspected of canL, and estimated the IFAT sensitivity at 89% and the specificity at 45% (the authors also refer to cross-reactions with *Ehrlichia canis*). Iniesta and colleagues [44] found a similar lack of specificity in IFAT, recording a very poor performance when having to discriminate between uninfected dogs and asymptomatic infected ones.

Parallel interpretation of the two test results yielded high prevalence estimates, as expected: 41% (Tlemcen)—62% (Bejaia) for the first survey period and 29% (Mostaganem)—52% (Bejaia) for the second, compared to the estimated 12% in Algiers [12]. As explained by [18], parallel interpretation increases the sensitivity of the combined tests, but decreases the specificity. Serial interpretation resulted in estimates of 8% (Mostaganem, Boumerdes) -15% (Bejaia) for the first period and 11% (Mostaganem)—31% (Bejaia) for the second, more in line with the earlier findings [12] and other countries: 33% in northern Spain [45] and 21% in north-eastern Portugal [46], where the authors adopted a parallel/serial hybrid interpretation (utilize three or four tests and consider an animal infected when at least two tests yield a positive result).

Different methods exist to estimating the sensitivity and specificity of diagnostic tests and disease prevalence in the absence of a gold standard, see e.g. [47]; [30]; [48]. This situation is invariably characterized by an underspecified model and it must be appreciated that the posterior estimates, resulting from these approaches, are always a combination of prior assumptions (prior information, prior constraints) and the data at hand, i.e. the prior assumptions are an inherent part of the final estimates [49]. As long as the model can be expressed as a combination of prior constraints and a model to describe the data, all approaches offer a methodology to assess the concordance between the prior information and the data within a Bayesian framework. In this study, the choice was made for the statistics developed and used by [30].

The first group of prior assumptions concerns the conditional independence between the tests: the probability that a truly infected dog tests positive in DAT is independent of the test result for IFAT (and vice-versa) and the probability that a truly non-infected dogs tests negative in DAT is independent of the test result for IFAT (and vice versa). This constraint is used by the so-called Hui-Walter approach. The results clearly showed that the data do not agree with this prior assumption (Table 5). For tests that measure similar biologic processes such as serum antibody responses to infectious agents, it is logical to expect that test results will be dependent, conditional on the animal’s true status [50]. This is the case for IFAT and DAT that both detect anti-Leishmania IgG [1].

When assuming conditional test dependence, but constant sensitivity and specificity within the four geographic group ×period combinations (Tlemcen and Mostaganem on the one hand, and Tipaza, Boumerdes, Bejaia and Jijel on the other ×resp. first and second period), the negative *p_D_* values also indicates a conflict between the prior assumptions (constancy of test characteristics within the group ×period combination) and the data.

Only the full model yields acceptable estimates, yielding correct *p_D_*, *DIC* and *Bayes-p* values. The prevalence estimates lie between 11% (Mostaganem, first period) and 38% (Bejaia, second period) (Table 6) and are in accordance with those obtained by [12] and are similar to the results obtained through the serial interpretation of the two test results (Table 3).

The results of the present study once more show that IFAT is far from a gold standard with sensitivity varying (in function of locality) between 86% and 88% and specificity between 65% and 87%. Rodriguez-Cortes and colleagues [51, 52] used an experimental infection with *L. infantum* as gold standard and obtained an estimated sensitivity of 63% (95% CI: 43-79%) and an estimated specificity of 82% (95% CI:72-99%) for IFAT.

DAT showed a low sensitivity in the present study, but a consistently high specificity (Table 6). In various studies involving dogs resident in areas endemic to VL, reviewed by [53], DAT was found to have a sensitivity ranging between 70% and 100% and a specificity between 85% and 100%.

The prevalence estimated for the two westernmost localities (Tlemcen and Mostaganem) does not exceed 15% and the highest values are found for Bejaia. The latter lies in the area of Kabylie, which is one of the main agricultural areas of the country because of its higher rainfall. In fact there is a general increasing trend in rainfall from west to east and a similar relationship between rainfall and seropositivity in dogs has also been noticed in Greece [39]. A concordance was also found between the present results and the incidence of human cases of visceral leishmaniasis, where a maximum was also recorded for Bejaia [16].

Sandflies (*Phlebotomus* spp.), the vectors of *L. infantum* in the mediterranean basin, are active during the warmer months of the year [54]. The present study thus involved sampling during two periods, one before the main vector season and one after. No difference was found between the two sampling periods in the two westernmost localities (Tlemcen and Mostaganem) and some indication for an increase from the first to the second sampling stage, although there was a considerable overlap between the posterior probability distributions. This finding is in agreement with the results obtained by [54], who also did not find an effect of sandfly season on the prevalence of *L. infantum* infection or parasite-specific immune responses.

On the other hand, a monthly serological follow up of antibody titres against *L. infantum* in hunting dogs in southern Spain showed a significant increase in the percentage of dogs with titres >1/160: from 12.1% in April to 19.2% in October [55].

To the best of our knowledge, this is the first large-scale study of canL in Algeria. It confirms the presence of CanL in several cities of the Algerian littoral from west to east and it demonstrates a concordance between the CanL prevalence and the incidence rate of the Human Visceral Leishmaniasis. It further highlights the perils when using IFAT as a gold standard for CanL diagnosis.

## Supporting Information

S1 Listing 1 Indices of positive and negative agreement and their confidence intervals. (PDF)

S2 Listing 2 Hui-Walter model estimation in a Bayesian framework (data shown for Tlemcen). (PDF)

S3 Listing 3 Test characteristics and CanL prevalence when localities are divided in two groups. (PDF)

S4 Listing 4 Test characteristics and CanL prevalence using full Bayesian model. (PDF)

## References

[1] Alvar J, Cañavate C, Molina R, Moreno J, Nieto J (2004) Canine leishmaniasis. Advances in Parasitology 57: 1–88. 10.1016/S0065-308X(04)57001-X 15504537

[2] Moreno J, Alvar J (2002) Canine leishmaniasis: epidemiological risk and the experimental model. Trends in Parasitology 18: 399–405. 10.1016/S1471-4922(02)02347-4 12377257

[3] Killick-Kendrick R (1990) Phlebotomine vectors of the leishmaniases: a review. Medical and Veterinary Entomology 4: 1–24. 10.1111/j.1365-2915.1990.tb00255.x 2132963

[4] Izri M, Belazzoug S, Boudjebla Y, Dereure J, Pratlong S, et al (1990) *Leishmania infantum* MON-1 isolated from *Phlebotomus perniciosus*, in Kabylia (Algeria). Annales de Parasitologie Humaine et Comparée 65: 151–152. 2080833

[5] Oliva G, Scalone A, Foglia-Manzillo V, Gramiccia M, Pagano A, et al (2006) Incidence and time course of *Leishmania infantum* infections examined by parasitological, serologic, and nested-PCR techniques in a cohort of naive dogs exposed to three consecutive transmission seasons. Journal of Clinical Microbiology 44: 1318–1322. 10.1128/JCM.44.4.1318-1322.2006 16597857 PMC1448675

[6] Killick-Kendrick R, Killick-Kendrick M, Tang Y (1995) Anthroponotic cutaneous leishmaniasis in Kabul, Afghanistan: the high susceptibility of *Phlebotomus sergenti* to *Leishmania tropica* . Transactions of the Royal Society of Tropical Medicine and Hygiene 89: 477 10.1016/0035-9203(95)90072-1 8560513

[7] Alvar J, Molina R, San Andrés M, Tesouro M, Nieto J, et al (1994) Canine leishmaniasis: clinical, parasitological and entomological follow-up after chemotherapy. Annals of Tropical Medicine and Parasitology 88: 371–378. 7979624 10.1080/00034983.1994.11812879

[8] Molina R, Amela C, Nieto J, San-Andres M, Gonzalez F, et al (1994) Infectivity of dogs naturally parasitized by *Leishmania infantum* to colonized *Phlebotomus perniciosus* . Transactions of the Royal Society of Tropical Medicine and Hygiene 88: 491–493. 10.1016/0035-9203(94)90446-4 7570854

[9] Scalone A, De Luna R, Oliva G, Baldi L, Satta G, et al (2002) Evaluation of the *Leishmania* recombinant k39 antigen as a diagnostic marker for canine leishmaniasis and validation of a standardized enzyme-linked immunosorbent assay. Veterinary Parasitology 104: 275–285. 10.1016/S0304-4017(01)00643-4 11836028

[10] Gomes A, Armelin I, Menon S, Pereira-Chioccola V (2008) *Leishmania (V.) braziliensis*: detection by PCR in biopsies from patients with cutaneous leishmaniasis. Experimental Parasitology 119: 319–324. 10.1016/j.exppara.2008.02.014 18442815

[11] OIE (2014). Leishmaniosis. URL http://www.oie.int/fileadmin/Home/eng/Health_standards/tahm/2.01.08_LEISHMANIOSIS.pdf

[12] Adel A, Saegerman C, Speybroeck N, Praet N, Victor B, et al (2010) Canine leishmaniasis in Algeria: True prevalence and diagnostic test characteristics in groups of dogs of different functional type. Veterinary Parasitology 172: 204–213. 10.1016/j.vetpar.2010.05.009 20627416

[13] Addadi K, Dedet JP (1976) Epidemiology of leishmaniasis in Algeria. 6. Survey of clinical cases of infantile visceral leishmaniasis from 1965 to 1974. Bulletin de la Société de Pathologie Exotique 69: 68–75. 1036477

[14] Belazzoug S, Addadi K, Mokrani T, Hafirassou N, Hamrioui B, et al (1985) Visceral leishmaniasis in Algeria: study of cases hospitalized between 1975 and 1984 [in French]. Annales de la Société Belge de Médecine Tropicale 65: 329–335. 4096557

[15] Harrat Z, Addadi K, Belkaïd M, Tabet-Derraz O (1992) Algérie Recensement des cas de leishmaniose (période 1985–1990). Bulletin de la Société de Pathologie Exotique 85: 296–301. 1446179

[16] Adel A, Boughoufalah A, Saegerman C, De Deken R, Bouchene Z, et al (2014) Epidemiology of visceral leishmaniasis in algeria: An update. PLoS One 9: e99207 10.1371/journal.pone.0099207 24949958 PMC4064973

[17] Bertrand P, Bénichou J, Grenier P, Chastang C (2005) Hui and Walter’s latent-class reference-free approach may be more useful in assessing agreement than diagnostic performance. Journal of Clinical Epidemiology 58: 688–700. 10.1016/j.jclinepi.2004.10.021 15939220

[18] Dohoo I, Martin W, Stryhn H (2003) Veterinary Epidemiologic Research. AVC Inc., Charlottetown.

[19] Collins J, Huynh M (2014) Estimation of diagnostic accuracy without full verification: a review of latent-class methods. Statistics in Medicine 33: 4141–4169. 10.1002/sim.6218 24910172 PMC4199084

[20] Hui S, Walter S (1980) Estimating the error rates of diagnostic tests. Biometrics 36: 167–171. 10.2307/2530508 7370371

[21] Dendukuri N, Joseph L (2001) Bayesian approaches to modelling the conditional dependence between multiple diagnostic tests. Biometrics 57: 208–217. 10.1111/j.0006-341X.2001.00158.x 11252592

[22] Vercammen F, De Deken R (1996) Kinetics during allopurinol treatment in canine leishmaniasis. Veterinary Record 139: 264 10.1136/vr.139.11.264-a 8888563

[23] Mancianti F, Meciani N (1988) Specific serodiagnosis of canine leishmaniasis by indirect immunofluorescence, indirect hemagglutination, and counterimmunoelectrophoresis. American Journal of Veterinary Research 49: 1409–1411. 3052194

[24] Abranches P, Lopes F, Silva F, Ribeiro M, Pires C (1983) Kala-azar in Portugal. iii. results of a survey on canine leishmaniasis performed in the Lisbon region. comparison of urban and rural zones. Annales de Parasitologie Humaine et Comparée 58: 307–315. 6357029

[25] Abranches P, Silva-Pereira M, Conceição Silva F, Santos-Gomes G, Janz J (1991) Canine leishmaniasis: pathological and ecological factors influencing transmission of infection. Journal of Parasitology 77: 557–561. 10.2307/3283159 1865262

[26] El Harith A, Slappendel R, Reiter I, Van Knapen F, De Korte P, et al (1989) Application of a Direct Agglutination Test for Detection of Specific Anti-*Leishmania* Antibodies in the Canine Reservoir. Journal of Clinical Microbiology 27: 2252–2257. 2685025 10.1128/jcm.27.10.2252-2257.1989 PMC267005

[27] Boelaert M, Aoun K, Liinev J, Goetghebeur E, Van Der Stuyft P (1999) The potential of latent class analysis in diagnostic test validation for canine *Leishmania infantum* infection. Epidemiology and Infection 123: 499–506. 10.1017/S0950268899003040 10694163 PMC2810786

[28] Cicchetti D, Feinstein A (1990) High agreement but low kappa: II. resolving the paradoxes. Journal of Clinical Epidemiology 43: 551–558. 10.1016/0895-4356(90)90159-M 2189948

[29] Graham P, Bull B (1998) Approximate standard errors and confidence intervals for indices of positive and negative agreement. Journal of Clinical Epidemiology 51: 763–771. 10.1016/S0895-4356(98)00048-1 9731925

[30] Berkvens D, Speybroeck N, Praet N, Adel A, Lesaffre E (2006) Estimating disease prevalence in a Bayesian framework using probabilistic constraints. Epidemiology 17: 145–153. 10.1097/01.ede.0000198422.64801.8d 16477254

[31] Spiegelhalter D, Thomas A, Best N, Lunn D (2003) WinBUGS Version 1.4 User Manual. MRC Biostatistics Unit, Cambridge.

[32] Rogan W, Gladen B (1978) Estimating prevalence from the results of a screening test. American Journal of Epidemiology 107: 71–76. 623091 10.1093/oxfordjournals.aje.a112510

[33] Diouani M, Alaya Bouafif N, Bettaib J, Ouzir H, Jedidi S, et al (2008) Dogs *L. infantum* infection from an endemic region of the north of Tunisia: a prospective study. Archives de L’institut Pasteur Tunis 85: 55–61. 19469416

[34] Zoghlami Z, Chouihi E, Barhoumi W, Dachraoui K, Massoudi N, et al. (2014) Interaction between canine and human visceral leishmaniases in a holoendemic focus of central Tunisia. 10.1016/j.actatropica.2014.06.012 25004438

[35] Nejjar R, Lemrani M, Malki A, Ibrahimy S, Amarouch H, et al (1998) Canine leishmaniasis due to *Leishmania infantum* MON-1 in northern Morocco. Parasite 5: 325–330. 10.1051/parasite/1998054325 9879555

[36] Rami M, Atarhouch T, Sabri M, Cadi Soussi M, Benazzou T, et al (2003) Canine leishmaniasis in the Rif mountains (Moroccan Mediterranean coast): a seroepidemiological survey. Parasite 10: 79–85. 10.1051/parasite/2003101p77 12669354

[37] Morillas F, Sanchez Rabasco F, Ocaña J, Martin-Sanchez J, Ocaña Wihelmi J, et al (1996) Leishmaniosis in the focus of the Axarquía region, Malaga province, southern spain: a survey of the human, dog, and vector. Parasitological Research 82: 569–570. 10.1007/s004360050164 8832741

[38] Zaffaroni E, Rubaudo L, Lanfranchi P, Mignone W (2014) Epidemiological patterns of canine leishmaniosis in Western Liguria (Italy). Veterinary Parasitology 81: 11–19. 10.1016/S0304-4017(98)00237-4 9950324

[39] Ntais P, Sifaki-Pistola D, Christodoulou V, Messaritakis I, Pratlong F, et al (2013) Leishmaniasis in Greece. American Journal of Tropical Medicine and Hygiene 89: 906–915. 10.4269/ajtmh.13-0070 24062479 PMC3820334

[40] Schallig H, Cardoso L, Semião Santos S (2013) Seroepidemiology of canineleishmaniosis in leishmaniosis in évora (southern Portugal): 20-year trends. Parasites and Vectors 6: 100 10.1186/1756-3305-6-100 23587181 PMC3640909

[41] Neogy A, Vouldoukis I, Silva O, Tsenlentis Y, Lascombe J, et al (1992) Serodiagnosis and screening of canine visceral leishmaniasis in an endemic area of Corsica: Applicability of a direct agglutination. American Journal of Tropical Medicine and Hygiene 47: 772–777. 1471734 10.4269/ajtmh.1992.47.772

[42] Ertabaklar H, Ozensoy Toz S, Ozkan A, Rastgeldi S, Cuneyt Balcioglu I, et al (2005) Serological and entomological survey in a zoonotic visceral leishmaniasis focus of North-Central Anatolia, Turkey: Corum province. Acta Tropica 93: 239–246. 10.1016/j.actatropica.2005.01.002 15716053

[43] Chaouch M, Mhadhbi M, Adams E, Schoone G, Limam S, et al (2013) Development and evaluation of a loop-mediated isothermal amplification assay for rapid detection of *Leishmania infantum* in canine leishmaniasis based on cysteineprotease B genes. Veterinary Parasitology 198: 78–84. 10.1016/j.vetpar.2013.07.038 23972768

[44] Iniesta L, Fernández-Barredo S, Bulle B, Gómez M, Piarroux R, et al (2002) Diagnostic Techniques to Detect Cryptic Leishmaniasis in Dogs. Clinical and Diagnostic Laboratory Immunology 9: 1137–1141. 10.1128/CDLI.9.5.1137-1141.2002 12204974 PMC120076

[45] Ballart C, Alcover M, Picado A, Nieto J, Castillejo S, et al (2013) First survey on canine leishmaniasis in a non classical area of the disease in Spain (Lleida, Catalonia) based on a veterinary questionnaire and a cross-sectional study. Preventive Veterinary Medecine 109: 116–127. 10.1016/j.prevetmed.2012.09.003 23022112

[46] Sousa S, Lopes A, Cardoso L, Silvestre L, Schallig H, et al (2011) Seroepidemiological survey of *Leishmania infantum* infection in dogs from northeastern Portugal. Acta Tropica 120: 82–87. 10.1016/j.actatropica.2011.06.003 21741348

[47] Enoë C, Georgiadis, MP J, WO (2000) Estimation of sensitivity and specificity of diagnostic tests and disease prevalence when the true diseases state is unknown. Preventive Veterinary Medicine 45: 61–81. 10.1016/S0167-5877(00)00117-3 10802334

[48] Dendukuri N, Hadgu A, Wang L (2009) Modeling conditional dependence between diagnostic tests: a multiple latent variable model. Statistics in Medicine 28: 441–461. 10.1002/sim.3470 19067379

[49] Lesaffre E, Speybroeck N, Berkvens D (2007) Bayes and diagnostic testing. Veterinary Parasitology 148: 58–61. 10.1016/j.vetpar.2007.05.010 17566663

[50] Gardner I, Stryhn H, Ind P, Collins M (2000) Conditional dependence between tests affects the diagnosis and surveillance of animal diseases. Preventive Veterinary Medecine 45: 107–122. 10.1016/S0167-5877(00)00119-7 10802336

[51] Rodríguez-Córtes A, Ojeda A, Francino O, López-Fuertes L, Timón M, et al (2010) *Leishmania* infection: Laboratory Diagnosing in the Absence of a “Gold Standard”. American Journal of Tropical Medicine and Hygiene 82: 251–256. 10.4269/ajtmh.2010.09-0366 20134001 PMC2813166

[52] Rodríguez-Córtes A, Ojeda A, Todolí F, Alberola J (2013) Performance of commercially available serological diagnostic tests to detect *Leishmania infantum* infection on experimentally infected dogs. Veterinary Parasitology 191: 363–366. 10.1016/j.vetpar.2012.09.009 23021261

[53] Maia C, Campino L (2008) Methods for diagnosis of canine leishmaniasis and immune response to infection. Veterinary Parasitology 158: 274–287. 10.1016/j.vetpar.2008.07.028 18789583

[54] Fernández-Bellon H, Solano-Gallego L, Rodríguez-Córtes A, Ferrer L, Gallego M, et al (2008) Little evidence of seasonal variation of natural infection by *Leishmania infantum* in dogs in Spain. Veterinary Parasitology 155: 32–36. 10.1016/j.vetpar.2008.04.009 18524491

[55] Acedo-Sánchez C, F MM, Sanchíz-Marín M, Martín-Sánchez J (1998) Changes in antibody titres against *Leishmania infantum* in naturally infected dogs in southern Spain. Veterinary Parasitology 75: 1–8. 10.1016/S0304-4017(97)00196-9 9566089

