# Supplementary material for: Estimation of Canine Leishmania Infection Prevalence in Six Cities of the Algerian Littoral Zone Using a Bayesian Approach
Source: PLoS One. 2015 Mar 20;10(3):e0117313. doi: 10.1371/journal.pone.0117313 (PMC4368835; doi:10.1371/journal.pone.0117313)
Supplement: S1 Listing 1 — (PDF) [file pone.0117313.s001.pdf]

S1 Listing 1

Listing 1: Indices of positive and negative agreement and their confidence intervals

```
# Individual localities first sampling round
GrahamBull(16,12,47,107)
GrahamBull(12,0,50,81)
GrahamBull(26,49,56,111)
GrahamBull(17,21,54,114)
GrahamBull(30,28,64,76)
GrahamBull(30,30,32,117)
#Total first sampling round
GrahamBull(131,140,303,606)
# Individual localities second sampling round
GrahamBull(16,13,29,94)
GrahamBull(19,3,27,120)
GrahamBull(50,19,17,123)
GrahamBull(24,7,17,59)
GrahamBull(63,11,32,97)
GrahamBull(40,7,21,96)
# Total second sampling round
GrahamBull(212,60,143,589)
# Grand total
GrahamBull(343,200,446,1195)
GrahamBull = function(a=1, b=1, c=1, d=1)
{
#               test 1
#              pos    neg
#          pos   a      b
#     test2
#          neg   c      d
alpha1 = 0.25
alpha2 = 0.25
alpha3 = 0.25
alpha4 = 0.25
K = 500
pi = rbeta(K, (a+alpha1), (b+c+d+alpha2+alpha3+alpha4))
be = rbeta(K, (d+alpha4), (b+c+alpha2+alpha3))
pi4 = (1-pi)*be
pos = (2*pi)/(pi+1-pi4)
neg = (2*pi4)/(pi4+1-pi)
diff = pos-neg
quapos = quantile(pos,c(.025,.975))
quaneg = quantile(neg,c(.025,.975))
quadiff = quantile(diff,c(.025,.975))
p1 = a/(a+b+c+d)
p2 = b/(a+b+c+d)
p3 = c/(a+b+c+d)
p4 = d/(a+b+c+d)
positivagre = 2*p1/(2*p1+p2+p3)
negativagre = 2*p4/(2*p4+p2+p3)
#####Results########
print(paste("p(+/+): ",p1),quote=F)
print(paste("p(+/-): ",p2),quote=F)
print(paste("p(-/+): ",p3),quote=F)
```

Listing 1 (Cont.): Indices of positive and negative agreement and their confidence intervals

```
print(paste("p(-/-): ",p4),quote=F)
print(paste("Positive agreement: ",positivagre),quote=F)
print(paste("Negative agreement: ",negativagre),quote=F)
print("95% CI positive agreement", quote=F)
print(quapos,digits=5)
print("95% CI negative agreement", quote=F)
print(quaneg,digits=5)
print("95% CI positive agreement - negative agreement", quote=F)
print(quadiff,digits=5)
}
```
