# Supplementary material for: Estimation of Canine Leishmania Infection Prevalence in Six Cities of the Algerian Littoral Zone Using a Bayesian Approach
Source: PLoS One. 2015 Mar 20;10(3):e0117313. doi: 10.1371/journal.pone.0117313 (PMC4368835; doi:10.1371/journal.pone.0117313)
Supplement: S2 Listing 2 — (PDF) [file pone.0117313.s002.pdf]

S2 Listing 2

Listing 2: Hui-Walter model estimation in a Bayesian framework (data shown for Tlemcen)

```
model
{
res1[1:4] ~ dmulti(p1[1:4], n1)
res2[1:4] ~ dmulti(p2[1:4], n2)
p1[1] <- th[1]*th[3]*th[4] + (1-th[1])*(1-th[5])*(1-th[6])
p1[2] <- th[1]*th[3]*(1-th[4]) + (1-th[1])*(1-th[5])*th[6]
p1[3] <- th[1]*(1-th[3])*th[4] + (1-th[1])*th[5]*(1-th[6])
p1[4] <- th[1]*(1-th[3])*(1-th[4]) + (1-th[1])*th[5]*th[6]
p2[1] <- th[2]*th[3]*th[4] + (1-th[2])*(1-th[5])*(1-th[6])
p2[2] <- th[2]*th[3]*(1-th[4]) + (1-th[2])*(1-th[5])*th[6]
p2[3] <- th[2]*(1-th[3])*th[4] + (1-th[2])*th[5]*(1-th[6])
p2[4] <- th[2]*(1-th[3])*(1-th[4]) + (1-th[2])*th[5]*th[6]
th[1] ~ dunif(0,0.5)
th[2] ~ dunif(0,0.5)
th[3] ~ dunif(0,1)
th[4] ~ dunif(0,1)
th[5] ~ dunif(0,1)
th[6] ~ dunif(0,1)
res3[1:4] ~ dmulti(p1[1:4], n1)
res4[1:4] ~ dmulti(p2[1:4], n2)
for (i in 1:4)
{
d1[i] <- res1[i]*log(max(res1[i],1)/(p1[i]*n1))
d2[i] <- res3[i]*log(max(res3[i],1)/(p1[i]*n1))
d3[i] <- res2[i]*log(max(res2[i],1)/(p2[i]*n2))
d4[i] <- res4[i]*log(max(res4[i],1)/(p2[i]*n2))
}
bayesp[1] <- step( sum(d1[]) - sum(d2[]) )
bayesp[2] <- step( sum(d3[]) - sum(d4[]) )
}
list(res1 = c(16, 47, 12, 107), n1 = 182 , res2 = c(16, 29, 13, 94),
    n2 = 152)
```
