# Supplementary material for: Estimation of Canine Leishmania Infection Prevalence in Six Cities of the Algerian Littoral Zone Using a Bayesian Approach
Source: PLoS One. 2015 Mar 20;10(3):e0117313. doi: 10.1371/journal.pone.0117313 (PMC4368835; doi:10.1371/journal.pone.0117313)
Supplement: S3 Listing 3 — (PDF) [file pone.0117313.s003.pdf]

S3 Listing 3

Listing 3: Test characteristics and CanL prevalence when localities are divided in two groups

```
model
{
res01[1:4] ~ dmulti(p01[1:4], n01)
res02[1:4] ~ dmulti(p02[1:4], n02)
res03[1:4] ~ dmulti(p03[1:4], n03)
res04[1:4] ~ dmulti(p04[1:4], n04)
res05[1:4] ~ dmulti(p05[1:4], n05)
res06[1:4] ~ dmulti(p06[1:4], n06)
res07[1:4] ~ dmulti(p07[1:4], n07)
res08[1:4] ~ dmulti(p08[1:4], n08)
res09[1:4] ~ dmulti(p09[1:4], n09)
res10[1:4] ~ dmulti(p10[1:4], n10)
res11[1:4] ~ dmulti(p11[1:4], n11)
res12[1:4] ~ dmulti(p12[1:4], n12)

p01[1] <- th[ 1]*   th[13] *   th[15]  + (1-th[ 1])*(1-th[14])*(1-th[18])
p01[2] <- th[ 1]*   th[13] *(1-th[15]) + (1-th[ 1])*(1-th[14])*   th[18]
p01[3] <- th[ 1]*(1-th[13])*   th[16]  + (1-th[ 1])*   th[14] *(1-th[17])
p01[4] <- th[ 1]*(1-th[13])*(1-th[16]) + (1-th[ 1])*   th[14] *   th[17]
p02[1] <- th[ 2]*   th[13] *   th[15]  + (1-th[ 2])*(1-th[14])*(1-th[18])
p02[2] <- th[ 2]*   th[13] *(1-th[15]) + (1-th[ 2])*(1-th[14])*   th[18]
p02[3] <- th[ 2]*(1-th[13])*   th[16]  + (1-th[ 2])*   th[14] *(1-th[17])
p02[4] <- th[ 2]*(1-th[13])*(1-th[16]) + (1-th[ 2])*   th[14] *   th[17]

p03[1] <- th[ 3]*   th[19] *   th[21]  + (1-th[ 3])*(1-th[20])*(1-th[24])
p03[2] <- th[ 3]*   th[19] *(1-th[21]) + (1-th[ 3])*(1-th[20])*   th[24]
p03[3] <- th[ 3]*(1-th[19])*   th[22]  + (1-th[ 3])*   th[20] *(1-th[23])
p03[4] <- th[ 3]*(1-th[19])*(1-th[22]) + (1-th[ 3])*   th[20] *   th[23]
p04[1] <- th[ 4]*   th[19] *   th[21]  + (1-th[ 4])*(1-th[20])*(1-th[24])
p04[2] <- th[ 4]*   th[19] *(1-th[21]) + (1-th[ 4])*(1-th[20])*   th[24]
p04[3] <- th[ 4]*(1-th[19])*   th[22]  + (1-th[ 4])*   th[20] *(1-th[23])
p04[4] <- th[ 4]*(1-th[19])*(1-th[22]) + (1-th[ 4])*   th[20] *   th[23]

p05[1] <- th[ 5]*   th[25] *   th[27]  + (1-th[ 5])*(1-th[26])*(1-th
```

Listing 3 (Cont.): Test characteristics and CanL prevalence when localities are divided in two groups

```
    [30])
p05[2] <- th[ 5]*   th[25] *(1-th[27]) + (1-th[ 5])*(1-th[26])*   th[30]
p05[3] <- th[ 5]*(1-th[25])*   th[28]  + (1-th[ 5])*   th[26] *(1-th[29])
p05[4] <- th[ 5]*(1-th[25])*(1-th[28]) + (1-th[ 5])*   th[26] *   th[29]
p06[1] <- th[ 6]*   th[25] *   th[27]  + (1-th[ 6])*(1-th[26])*(1-th[30])
p06[2] <- th[ 6]*   th[25] *(1-th[27]) + (1-th[ 6])*(1-th[26])*   th[30]
p06[3] <- th[ 6]*(1-th[25])*   th[28]  + (1-th[ 6])*   th[26] *(1-th[29])
p06[4] <- th[ 6]*(1-th[25])*(1-th[28]) + (1-th[ 6])*   th[26] *   th[29]
p07[1] <- th[ 7]*   th[25] *   th[27]  + (1-th[ 7])*(1-th[26])*(1-th[30])
p07[2] <- th[ 7]*   th[25] *(1-th[27]) + (1-th[ 7])*(1-th[26])*   th[30]
p07[3] <- th[ 7]*(1-th[25])*   th[28]  + (1-th[ 7])*   th[26] *(1-th[29])
p07[4] <- th[ 7]*(1-th[25])*(1-th[28]) + (1-th[ 7])*   th[26] *   th[29]
p08[1] <- th[ 8]*   th[25] *   th[27]  + (1-th[ 8])*(1-th[26])*(1-th[30])
p08[2] <- th[ 8]*   th[25] *(1-th[27]) + (1-th[ 8])*(1-th[26])*   th[30]
p08[3] <- th[ 8]*(1-th[25])*   th[28]  + (1-th[ 8])*   th[26] *(1-th[29])
p08[4] <- th[ 8]*(1-th[25])*(1-th[28]) + (1-th[ 8])*   th[26] *   th[29]

p09[1] <- th[ 9]*   th[31] *   th[33]  + (1-th[ 9])*(1-th[32])*(1-th[36])
p09[2] <- th[ 9]*   th[31] *(1-th[33]) + (1-th[ 9])*(1-th[32])*   th[36]
p09[3] <- th[ 9]*(1-th[31])*   th[34]  + (1-th[ 9])*   th[32] *(1-th[35])
p09[4] <- th[ 9]*(1-th[31])*(1-th[34]) + (1-th[ 9])*   th[32] *   th[35]
p10[1] <- th[10]*   th[31] *   th[33]  + (1-th[10])*(1-th[32])*(1-th[36])
p10[2] <- th[10]*   th[31] *(1-th[33]) + (1-th[10])*(1-th[32])*   th[36]
p10[3] <- th[10]*(1-th[31])*   th[34]  + (1-th[10])*   th[32] *(1-th[35])
p10[4] <- th[10]*(1-th[31])*(1-th[34]) + (1-th[10])*   th[32] *   th[35]
p11[1] <- th[11]*   th[31] *   th[33]  + (1-th[11])*(1-th[32])*(1-th[36])
p11[2] <- th[11]*   th[31] *(1-th[33]) + (1-th[11])*(1-th[32])*   th[36]
p11[3] <- th[11]*(1-th[31])*   th[34]  + (1-th[11])*   th[32] *(1-th[35])
p11[4] <- th[11]*(1-th[31])*(1-th[34]) + (1-th[11])*   th[32] *   th[35]
```

Listing 3 (Cont.): Test characteristics and CanL prevalence when localities are divided in two groups

```
p12[1] <- th[12]*   th[31] *   th[33]  + (1-th[12])*(1-th[32])*(1-th[36])
p12[2] <- th[12]*   th[31] *(1-th[33]) + (1-th[12])*(1-th[32])*   th[36]
p12[3] <- th[12]*(1-th[31])*   th[34]  + (1-th[12])*   th[32] *(1-th[35])
p12[4] <- th[12]*(1-th[31])*(1-th[34]) + (1-th[12])*   th[32] *   th[35]

th[ 1] ~ dunif(0, 0.5)
th[ 2] ~ dunif(0, 0.5)
th[ 3] ~ dunif(0, 0.5)
th[ 4] ~ dunif(0, 0.5)
th[ 5] ~ dunif(0, 0.5)
th[ 6] ~ dunif(0, 0.5)
th[ 7] ~ dunif(0, 0.5)
th[ 8] ~ dunif(0, 0.5)
th[ 9] ~ dunif(0, 0.5)
th[10] ~ dunif(0, 0.5)
th[11] ~ dunif(0, 0.5)
th[12] ~ dunif(0, 0.5)
th[13] ~ dunif(0, 1)
th[14] ~ dunif(0, 1)
th[15] ~ dunif(0, 1)
th[16] ~ dunif(0, 1)
th[17] ~ dunif(0, 1)
th[18] ~ dunif(0, 1)
th[19] ~ dunif(0, 1)
th[20] ~ dunif(0, 1)
th[21] ~ dunif(0, 1)
th[22] ~ dunif(0, 1)
th[23] ~ dunif(0, 1)
th[24] ~ dunif(0, 1)
th[25] ~ dunif(0, 1)
th[26] ~ dunif(0, 1)
th[27] ~ dunif(0, 1)
th[28] ~ dunif(0, 1)
th[29] ~ dunif(0, 1)
th[30] ~ dunif(0, 1)
th[31] ~ dunif(0, 1)
th[32] ~ dunif(0, 1)
th[33] ~ dunif(0, 1)
th[34] ~ dunif(0, 1)
th[35] ~ dunif(0, 1)
th[36] ~ dunif(0, 1)

res13[1:4] ~ dmulti(p01[1:4], n01)
res14[1:4] ~ dmulti(p02[1:4], n02)
res15[1:4] ~ dmulti(p03[1:4], n03)
res16[1:4] ~ dmulti(p04[1:4], n04)
res17[1:4] ~ dmulti(p05[1:4], n05)
res18[1:4] ~ dmulti(p06[1:4], n06)
res19[1:4] ~ dmulti(p07[1:4], n07)
res20[1:4] ~ dmulti(p08[1:4], n08)
res21[1:4] ~ dmulti(p09[1:4], n09)
res22[1:4] ~ dmulti(p10[1:4], n10)
```

Listing 3 (Cont.): Test characteristics and CanL prevalence when localities are divided in two groups

```
res23[1:4] ~ dmulti(p11[1:4], n11)
res24[1:4] ~ dmulti(p12[1:4], n12)

for (i in 1:4)
{
d01[i] <- res01[i]*log(max(res01[i],1)/(p01[i]*n01))
d13[i] <- res13[i]*log(max(res13[i],1)/(p01[i]*n01))
d02[i] <- res02[i]*log(max(res02[i],1)/(p02[i]*n02))
d14[i] <- res14[i]*log(max(res14[i],1)/(p02[i]*n02))
d03[i] <- res03[i]*log(max(res03[i],1)/(p03[i]*n03))
d15[i] <- res15[i]*log(max(res15[i],1)/(p03[i]*n03))
d04[i] <- res04[i]*log(max(res04[i],1)/(p04[i]*n04))
d16[i] <- res16[i]*log(max(res16[i],1)/(p04[i]*n04))
d05[i] <- res05[i]*log(max(res05[i],1)/(p05[i]*n05))
d17[i] <- res17[i]*log(max(res17[i],1)/(p05[i]*n05))
d06[i] <- res06[i]*log(max(res06[i],1)/(p06[i]*n06))
d18[i] <- res18[i]*log(max(res18[i],1)/(p06[i]*n06))
d07[i] <- res07[i]*log(max(res07[i],1)/(p07[i]*n07))
d19[i] <- res19[i]*log(max(res19[i],1)/(p07[i]*n07))
d08[i] <- res08[i]*log(max(res08[i],1)/(p08[i]*n08))
d20[i] <- res20[i]*log(max(res20[i],1)/(p08[i]*n08))
d09[i] <- res09[i]*log(max(res09[i],1)/(p09[i]*n09))
d21[i] <- res21[i]*log(max(res21[i],1)/(p09[i]*n09))
d10[i] <- res10[i]*log(max(res10[i],1)/(p10[i]*n10))
d22[i] <- res22[i]*log(max(res22[i],1)/(p10[i]*n10))
d11[i] <- res11[i]*log(max(res11[i],1)/(p11[i]*n11))
d23[i] <- res23[i]*log(max(res23[i],1)/(p11[i]*n11))
d12[i] <- res12[i]*log(max(res12[i],1)/(p12[i]*n12))
d24[i] <- res24[i]*log(max(res24[i],1)/(p12[i]*n12))
}
bayesp[ 1] <- step( sum(d01[]) - sum(d13[]) )
bayesp[ 2] <- step( sum(d02[]) - sum(d14[]) )
bayesp[ 3] <- step( sum(d03[]) - sum(d15[]) )
bayesp[ 4] <- step( sum(d04[]) - sum(d16[]) )
bayesp[ 5] <- step( sum(d05[]) - sum(d17[]) )
bayesp[ 6] <- step( sum(d06[]) - sum(d18[]) )
bayesp[ 7] <- step( sum(d07[]) - sum(d19[]) )
bayesp[ 8] <- step( sum(d08[]) - sum(d20[]) )
bayesp[ 9] <- step( sum(d09[]) - sum(d21[]) )
bayesp[10] <- step( sum(d10[]) - sum(d22[]) )
bayesp[11] <- step( sum(d11[]) - sum(d23[]) )
bayesp[12] <- step( sum(d12[]) - sum(d24[]) )

pr[ 1] <- th[ 1]
pr[ 2] <- th[ 2]
pr[ 3] <- th[ 3]
pr[ 4] <- th[ 4]
pr[ 5] <- th[ 5]
pr[ 6] <- th[ 6]
pr[ 7] <- th[ 7]
pr[ 8] <- th[ 8]
pr[ 9] <- th[ 9]
pr[10] <- th[10]
pr[11] <- th[11]
pr[12] <- th[12]

```

Listing 3 (Cont.): Test characteristics and CanL prevalence when localities are divided in two groups

```
se1[1]  <-  th[13]
se1[2]  <-  th[19]
se1[3]  <-  th[25]
se1[4]  <-  th[31]
sp1[1]  <-  th[14]
sp1[2]  <-  th[20]
sp1[3]  <-  th[26]
sp1[4]  <-  th[32]

se2[1]  <-  th[13]*th[15]+(1-th[13])*th[16]
se2[2]  <-  th[19]*th[21]+(1-th[19])*th[22]
se2[3]  <-  th[25]*th[27]+(1-th[25])*th[28]
se2[4]  <-  th[31]*th[33]+(1-th[31])*th[34]
sp2[1]  <-  th[14]*th[17]+(1-th[14])*th[18]
sp2[2]  <-  th[20]*th[23]+(1-th[20])*th[24]
sp2[3]  <-  th[26]*th[29]+(1-th[26])*th[30]
sp2[4]  <-  th[32]*th[35]+(1-th[32])*th[36]
}

list(res01 = c(16,47,12,107), n01 = 182,
res02 = c(12,50,0,81)   , n02 = 143,
res03 = c(16,29,13,94)  , n03 = 152,
res04 = c(19,27,3,120)  , n04 = 169,
res05 = c(26,56,49,111), n05 = 242,
res06 = c(17,54,21,114), n06 = 206,
res07 = c(30,64,28,76)  , n07 = 198,
res08 = c(30,32,30,117), n08 = 209,
res09 = c(50,17,19,123), n09 = 209,
res10 = c(24,17,7,59)   , n10 = 107,
res11 = c(63,32,11,97)  , n11 = 203,
res12 = c(40,21,7,96)   , n12 = 164)
```
