# Supplementary material for: Estimation of Canine Leishmania Infection Prevalence in Six Cities of the Algerian Littoral Zone Using a Bayesian Approach
Source: PLoS One. 2015 Mar 20;10(3):e0117313. doi: 10.1371/journal.pone.0117313 (PMC4368835; doi:10.1371/journal.pone.0117313)
Supplement: S4 Listing 4 — (PDF) [file pone.0117313.s004.pdf]

S4 Listing 4

Listing 4: Test characteristics and CanL prevalence using full Bayesian model

```
model
{
  for(i in 1:2)
  {
    for (j in 1:6)
      {
      pr[i,j]     ~ dunif(0, 1)
      se1[i,j]    ~ dunif(0.804, 0.997)
      se2[i,j]    ~ dunif(0.344, 0.961)
      sp1[i,j]    ~ dunif(0.602, 0.990)
      sp2[i,j]    ~ dunif(0.785, 0.990)
      covse[i,j] ~ dunif(-1, 1)
      covsp[i,j] ~ dunif(-1, 1)
      r[i,j,1:4] ~ dmulti(p[i,j,1:4], N[i,j])
      pos[i,j,1] <-    se1[i,j]   *    se2[i,j]   + covse[i,j]
      neg[i,j,1] <- (1-sp1[i,j]) * (1-sp2[i,j]) + covsp[i,j]
      pos[i,j,2] <-    se1[i,j]   * (1-se2[i,j]) - covse[i,j]
      neg[i,j,2] <- (1-sp1[i,j]) *    sp2[i,j]   - covsp[i,j]
      pos[i,j,3] <- (1-se1[i,j]) *    se2[i,j]   - covse[i,j]
      neg[i,j,3] <-    sp1[i,j]   * (1-sp2[i,j]) - covsp[i,j]
      pos[i,j,4] <- (1-se1[i,j]) * (1-se2[i,j]) + covse[i,j]
      neg[i,j,4] <-    sp1[i,j]   *    sp2[i,j]   + covsp[i,j]
      p[i,j,1] <- pr[i,j] * pos[i,j,1] + (1-pr[i,j]) * neg[i,j,1]
      p[i,j,2] <- pr[i,j] * pos[i,j,2] + (1-pr[i,j]) * neg[i,j,2]
      p[i,j,3] <- pr[i,j] * pos[i,j,3] + (1-pr[i,j]) * neg[i,j,3]
      p[i,j,4] <- pr[i,j] * pos[i,j,4] + (1-pr[i,j]) * neg[i,j,4]
      r2[i,j,1:4] ~ dmulti(p[i,j, 1:4],N[i,j])
      for ( k in 1:4)
      {
        d[i,j,k] <- r[i,j,k]*log(max(r[i,j,k],1)/(p[i,j,k]*N[i,j]))
        d2[i,j,k] <- r2[i,j,k]*log(max(r2[i,j,k],1)/(p[i,j,k]*N[i,j]))
        ## p > 0
        constraint1[i,j,k] <- step(p[i,j,k])
        O1[i,j,k] ~ dbern(constraint1[i,j,k])
        O1[i,j,k] <- 1

        ## p < 1
        constraint2[i,j,k] <- step(p[i,j,k] - 1)
        O2[i,j,k] ~ dbern(constraint2[i,j,k])
        O2[i,j,k] <- 0

        ## pos > 0
        constraint3[i,j,k] <- step(pos[i,j,k])
        O3[i,j,k] ~ dbern(constraint3[i,j,k])
        O3[i,j,k] <- 1

        ## pos < 1
        constraint4[i,j,k] <- step(pos[i,j,k] - 1)
        O4[i,j,k] ~ dbern(constraint4[i,j,k])
        O4[i,j,k] <- 0
```

Listing 4 (Cont.): Test characteristics and CanL prevalence using full Bayesian model

```

        ## neg > 0
        constraint5[i,j,k] <- step(neg[i,j,k])
        O5[i,j,k] ~ dbern(constraint5[i,j,k])
        O5[i,j,k] <- 1

        ## prob_sp < 1
        constraint6[i,j,k] <- step(neg[i,j,k] - 1)
        O6[i,j,k] ~ dbern(constraint6[i,j,k])
        O6[i,j,k] <- 0

        }
      bayesp[i,j] <- step(sum(d[i,j,1:4]) - sum(d2[i,j,1:4]))
      }
    }
  # differences
  for (j11 in 1:5)
  {
      for (j12 in (j11+1):6)
      {
          diff[j11,j12] <- pr[1,j11] - pr[1,j12]
      }
  }
  for (j23 in 1:6)
  {
      for (j33 in 1:6)
      {
          diff[j23,(j33+6)] <- pr[1,j23] - pr[2,j33]
      }
  }
     for (j21 in 1:5)
  {
    for (j22 in (j21+1):6)
    {
        diff[(j21+6),(j22+6)] <- pr[2,j21] - pr[2,j22]
    }
  }
  }

## DATA
list(r=structure(.Data=c(16,47,12,107,
12,50,0,81,
26,56,49,111,
17,54,21,114,
30,64,28,76,
30,32,30,117,
16,29,13,94,
19,27,3,120,
50,17,19,123,
24,17,7,59,
63,32,11,97,
40,21,7,96),
.Dim=c(2,6,4)),
N=structure(.Data=c(182,143,242,206,198,209,
152,169,209,107,203,164),
.Dim=c(2,6)))
```

Listing 4 (Cont.): Test characteristics and CanL prevalence using full Bayesian model

```

## INITS
list(r2=structure(.Data=c(16, 47, 12, 107, 12, 50, 0, 81, 26, 56, 49,
    111, 17, 54, 21, 114, 30, 64, 28, 76, 30, 32, 30, 117, 16, 29,
   13, 94, 19, 27, 3, 120, 50, 17, 19, 123, 24, 17, 7, 59, 63, 32,
   11, 97, 40, 21, 7, 96),.Dim=c(2,6,4)), pr=structure(.Data=c
   (0.5,0.5,0.5,0.5,0.5,0.5,0.5,0.5,0.5,0.5,0.5,0.5), .Dim=c(2,6)),
   se1=structure(.Data=c( 0.995 , 0.995 , 0.995 , 0.995 , 0.995 ,
   0.995 , 0.995 , 0.995 , 0.995 , 0.995 , 0.995 , 0.995 ), .Dim=c
   (2,6)), se2=structure(.Data=c( 0.9 , 0.9 , 0.9 , 0.9 , 0.9 , 0.9
   , 0.9 , 0.9 , 0.9 , 0.9 , 0.9 , 0.9 ), .Dim=c(2,6)), sp1=
   structure(.Data=c( 0.89 , 0.89 , 0.89 , 0.89 , 0.89 , 0.89 , 0.89
    , 0.89 , 0.89 , 0.89 , 0.89 , 0.89 ), .Dim=c(2,6)), sp2=
   structure(.Data=c( 0.97 , 0.97 , 0.97 , 0.97 , 0.97 , 0.97 , 0.97
    , 0.97 , 0.97 , 0.97 , 0.97 , 0.97 ), .Dim=c(2,6)), covse=
   structure(.Data=c( 0.002 , 0.002 , 0.002 , 0.002 , 0.002 , 0.002
   , 0.002 , 0.002 , 0.002 , 0.002 , 0.002 , 0.002), .Dim=c(2,6)),
   covsp=structure(.Data=c( 0.0057 , 0.0057 , 0.0057 , 0.0057 ,
   0.0057 , 0.0057 , 0.0057 , 0.0057 , 0.0057 , 0.0057 , 0.0057 ,
   0.0057 ), .Dim=c(2,6)))
list(r2=structure(.Data=c(16, 47, 12, 107, 12, 50, 0, 81, 26, 56, 49,
    111, 17, 54, 21, 114, 30, 64, 28, 76, 30, 32, 30, 117, 16, 29,
   13, 94, 19, 27, 3, 120, 50, 17, 19, 123, 24, 17, 7, 59, 63, 32,
   11, 97, 40, 21, 7, 96), .Dim=c(2,6,4)), pr=structure(.Data=c
   (0.5,0.5,0.5,0.5,0.5,0.5,0.5,0.5,0.5,0.5,0.5,0.5), .Dim=c(2,6)),
   se1=structure(.Data=c( 0.95 , 0.95 , 0.95 , 0.95 , 0.95 , 0.95 ,
   0.95 , 0.95 , 0.95 , 0.95 , 0.95 , 0.95 ), .Dim=c(2,6)), se2=
   structure(.Data=c( 0.8 , 0.8 , 0.8 , 0.8 , 0.8 , 0.8 , 0.8 , 0.8
   , 0.8 , 0.8 , 0.8 , 0.8 ), .Dim=c(2,6)), sp1=structure(.Data=c(
   0.75 , 0.75 , 0.75 , 0.75 , 0.75 , 0.75 , 0.75 , 0.75 , 0.75 ,
   0.75 , 0.75 , 0.75 ), .Dim=c(2,6)), sp2=structure(.Data=c( 0.895
   , 0.895 , 0.895 , 0.895 , 0.895 , 0.895 , 0.895 , 0.895 , 0.895 ,
    0.895 , 0.895 , 0.895 ), .Dim=c(2,6)), covse=structure(.Data=c(
   0.015 , 0.015 , 0.015 , 0.015 , 0.015 , 0.015 , 0.015 , 0.015 ,
   0.015 , 0.015 , 0.015 , 0.015 ), .Dim=c(2,6)), covsp=structure(.
   Data=c( 0.01 , 0.01 , 0.01 , 0.01 , 0.01 , 0.01 , 0.01 , 0.01 ,
   0.01 , 0.01 , 0.01 , 0.01 ), .Dim=c(2,6)))
list(r2=structure(.Data=c(16, 47, 12, 107, 12, 50, 0, 81, 26, 56, 49,
    111, 17, 54, 21, 114, 30, 64, 28, 76, 30, 32, 30, 117, 16, 29,
   13, 94, 19, 27, 3, 120, 50, 17, 19, 123, 24, 17, 7, 59, 63, 32,
   11, 97, 40, 21, 7, 96), .Dim=c(2,6,4)), pr=structure(.Data=c
   (0.5,0.5,0.5,0.5,0.5,0.5,0.5,0.5,0.5,0.5,0.5,0.5), .Dim=c(2,6)),
   se1=structure(.Data=c( 0.91 , 0.91 , 0.91 , 0.91 , 0.91 , 0.91 ,
   0.91 , 0.91 , 0.91 , 0.91 , 0.91 , 0.91 ), .Dim=c(2,6)), se2=
   structure(.Data=c( 0.8 , 0.8 , 0.8 , 0.8 , 0.8 , 0.8 , 0.8 , 0.8
   , 0.8 , 0.8 , 0.8 , 0.8 ), .Dim=c(2,6)), sp1=structure(.Data=c(
   0.85 , 0.85 , 0.85 , 0.85 , 0.85 , 0.85 , 0.85 , 0.85 , 0.85 ,
   0.85 , 0.85 , 0.85 ), .Dim=c(2,6)), sp2=structure(.Data=c( 0.9 ,
   0.9 , 0.9 , 0.9 , 0.9 , 0.9 , 0.9 , 0.9 , 0.9 , 0.9 , 0.9 , 0.9 )
   , .Dim=c(2,6)), covse=structure(.Data=c( 0.027 , 0.027 , 0.027 ,
   0.027 , 0.027 , 0.027 , 0.027 , 0.027 , 0.027 , 0.027 , 0.027 ,
   0.027 ), .Dim=c(2,6)), covsp=structure(.Data=c( 0.015 , 0.015 ,
   0.015 , 0.015 , 0.015 , 0.015 , 0.015 , 0.015 , 0.015 , 0.015 ,
   0.015 , 0.015 ), .Dim=c(2,6)))
```
